# MRI Brain Images Healthy and Pathological Tissues Classification with the Aid of Improved Particle Swarm Optimization and Neural Network

**DOI:** 10.1155/2015/807826

**Published:** 2015-04-22

**Authors:** V. Sheejakumari, B. Sankara Gomathi

**Affiliations:** ^1^Department of Information Technology, Rajaas Engineering College, Tirunelveli, Vadakkangulam, Tamil Nadu 627116, India; ^2^Department of Electronics & Instrumentation Engineering, National Engineering College, Kovilpatti, Tamil Nadu 628503, India

## Abstract

The advantages of magnetic resonance imaging (MRI) over other diagnostic imaging modalities are its higher spatial resolution and its better discrimination of soft tissue. In the previous tissues classification method, the healthy and pathological tissues are classified from the MRI brain images using HGANN. But the method lacks sensitivity and accuracy measures. The classification method is inadequate in its performance in terms of these two parameters. So, to avoid these drawbacks, a new classification method is proposed in this paper. Here, new tissues classification method is proposed with improved particle swarm optimization (IPSO) technique to classify the healthy and pathological tissues from the given MRI images. Our proposed classification method includes the same four stages, namely, tissue segmentation, feature extraction, heuristic feature selection, and tissue classification. The method is implemented and the results are analyzed in terms of various statistical performance measures. The results show the effectiveness of the proposed classification method in classifying the tissues and the achieved improvement in sensitivity and accuracy measures. Furthermore, the performance of the proposed technique is evaluated by comparing it with the other segmentation methods.

## 1. Introduction

Normally, in brain tissue segmentation on magnetic resonance (MR) images, the type of tissue present for each pixel or voxel in a 2D or 3D dataset, respectively, is determined based on the information captured from both MR images and prior knowledge of the brain. Segmentation of brain tissue in MRI is a crucial preprocessing step in several medical research and clinical applications, involving measurement of tissue volume, visualization, and analysis of anatomical structures, multimodality fusion and registration, functional brain mapping, pathology recognition, surgical planning and navigation, and brain substructure segmentation [1]. The investigations of medical images for computer-aided diagnosis and treatment often require segmentation as an initial stage. Medical image segmentation is a difficult and challenging task mainly due to the vague nature of the images [2–4]. Magnetic resonance imaging (MRI) is one of the most significant diagnostic imaging techniques, often used for the early detection of anomalous changes in tissues and organs [5, 6], and also it allows a radiologist to produce an image covering the internal features of living tissue because it is a noninvasive imaging technique [7].

It is well-known that the brain has a complicated structure; thus, accurate segmentation of brain is very decisive for detecting tumors, edema, necrotic tissues, white matter, gray matter, cerebrospinal fluid (CSF), or vasculature in order to provide proper treatment [8]. A technique to segment tissues into these categories is a vital step in quantitative morphology of brain because most brain structures are defined by boundaries of these tissue classes [9]. Unlike other diagnostic methods, magnetic resonance imaging (MRI) systems create numerous images, where diverse fundamental parameters of internal anatomical structures in the same body section are highlighted by each image with multiple contrasts, based on local variations of spin-spin relaxation time (T2), spin-lattice relaxation time (T1), and proton density (PD) [10]. The major obstruction to segmentation of MR images is the occurrence of noise, flaws in the scanners, and the structural variations of the imaging objects, which can be classified into four types, namely, thermal/electronic noise, magnetic field in homogeneities, biological tissue variations, and partial volume effects [11].

Furthermore, manual detection and analysis of lesions from MR brain images are normally time-consuming and expensive and can produce unacceptably high intraobserver and interobserver variability [12]. The efficacy of segmented MR images in the medical diagnostic process depends on the combination of two, often conflicting, requirements, that is, the removal of superfluous information present in the original MR images and the maintenance of significant information in the resulting segmented images [13, 14]. MR image segmentation methods are often analyzed in terms of their potentiality to discriminate (i) between cerebrospinal fluid (CSF), white matter, and gray matter and (ii) between normal tissues and abnormalities [15]. Recently, numerous techniques have been proposed for the segmentation of brain tissues in MR image. Some of them are classical pattern recognition techniques, rule-based systems, image analysis methods, crisp and fuzzy clustering procedures, feed-forward neural networks, fuzzy reasoning [16], geometric models to specify lesion boundaries, connected component analysis, deterministic annealing, atlas based techniques, and contouring approaches [17, 18].

## 2. Related Works

A few recent works related to this existing in the literature are reviewed in the following section.

Wang and Chen [19] have proposed a classification technique called vector seeded region growing (VSRG), in which the seed pixel vectors have been selected through standard deviation and relative Euclidean distance. By the VSRG processing, the data dimensionality of MRI has been reduced. A series of experiments has been carried out and compared to the normally used *c*-means technique for performance evaluation. The results have exhibited the efficacy of the proposed technique in MR image classification.

An automatic approach for the segmentation of anatomical 3D brain MR images has been proposed by Cherradi et al. [20] as well. The proposed technique includes many important steps. Initially, noise reduction has been done via median filtering. Secondly, segmentation of brain/nonbrain tissue has been carried out using a threshold morphologic brain extraction technique (TMBE). Subsequently, initial centroids estimation by gray level histogram analysis has been executed, and this stage yields to a modified version of fuzzy *C*-means algorithm (MFCM) that has been employed for MRI tissue segmentation. At last, 3D visualization of three clusters such as CSF, GM, and WM has been performed. The competency of the proposed technique has been validated by conducting wide segmentation experiments using simulated and real MR images. A confrontation of the technique with similar methods of the literature has been undertaken through diverse performance measures. The MFCM for tissue segmentation has achieved a gain in rapidity of convergence of about 70%.

Rajendran and Dhanasekaran [21] have investigated the segmentation of MRI brain image into different tissue types on brain image via possibilistic fuzzy *c*-means (PFCM) clustering algorithm. Application of this technique to MRI brain image has produced better segmentation result than the fuzzy *c*-mean (FCM) and fuzzy possibilistic *c*-means (FPCM) algorithms. The outcomes have been verified quantitatively through similarity metrics, false positive volume function (FPVF), and false negative volume functions (FNVF). These values have proved that the proposed PFCM has segmented the tumor class successfully by employing the membership and possibility (typicality) functions.

Mehta et al. [22] have presented an approach to automatically generate fuzzy rules for tissue classification in MRI. The proposed scheme was based on hybrid approach of two popular genetic algorithm based machine learning (GBML) techniques, Michigan and Pittsburg approach. The proposed approach has utilized a training dataset generated from manual segmented images with the aid of an expert in MRI. Features from image histogram and spatial neighborhood of pixels have been employed in fuzzy rules. The approach has been tested for classifying brain T2 weighted 2D axial images obtained by different pulse sequences into three main tissue types, namely, white matter (WM), gray matter (GM), and cerebrospinal fluid (CSF). Also, the experts have matched the results of proposed approach with manual segmentation. Moreover, it has been found that the performance of proposed approach was comparable.

Hussain et al. [23] have devised a technique for an accurate segmentation of normal and pathological tissues in the MRI brain images. In the proposed segmentation technique, initially the classification process has been done using fuzzy inference system (FIS) and FFBNN. Both classifiers have used the extracted image features as an input for the classification process. The features have been extorted in two ways from the MRI brain images. The FIS perform the classification process by generating the fuzzy rules using the extracted features. Five features have been extracted from the MRI images: two dynamic statistical features and three 2D wavelet decomposition features. In segmentation, the normal tissues such as WM, GM, and CSF have been segmented from the normal MRI images and pathological tissues such as edema and tumor have been segmented from the anomalous images. In the preprocessing stage, the noncortical tissues in the normal images have been removed. The implementation results have exhibited the potency of the proposed tissue segmentation technique in segmenting the tissues precisely from the MRI images. Moreover, the performance of the segmentation technique has been analyzed using performance measures such as accuracy, specificity, and sensitivity and compared with *K*-means clustering and fuzzy ANN based segmentation techniques.

In the previous tissues classification method, the healthy and pathological tissues are classified from the MRI brain images using HGANN (Figure 2) [24]. When compared to other tissues classification methods, this method performance lacks the accuracy and sensitivity measures. The classification method is inadequate in its performance in terms of these two parameters. So, to avoid these drawbacks, a new classification method with improved PSO technique is proposed in this paper. The outline of the paper is as follows. The proposed ISPONN classification process is briefly explained in Section 3. In Section 3.1, tissue segmentation process is performed and feature extraction process is described in Section 3.2. Sections 3.3 and 3.4 explain the heuristic feature selection by ISPO and tissue classification process. The experimental result and conclusion of this paper are given in Sections 4 and 5.

## 3. The Proposed IPSONN Technique for MRI Brain Tissues Classification

In this paper, we proposed an efficient method to classify the normal and pathological tissues in the MRI brain images with IPSO (improved particle swarm optimization) and FFBNN. Four major stages are involved in our proposed methodology:

- tissue segmentation;
- feature extraction;
- heuristic features selection by IPSO;
- tissue classification by FFBNN.

### 3.1. Tissue Segmentation

In training images, the tissues are segmented manually by MRI experts. Before the segmentation process, the input images are given to the preprocessing stage. In preprocessing, skull stripping method, described in [23], is applied to the input images in order to remove the distinct dark ring surrounding the brain tissues. After the skull stripping, the brain image tissues are segmented. The normal image obtained after skull stripping is denoted by *I*_*s*_.

#### 3.1.1. Normal Tissue Segmentation

In MRI brain images, the normal tissues such as WM, GM, and CSF are segmented. The detail description about this segmentation process is given in [23]. The major step is to segment the WM and GM tissues from the image *I*_*s*_ by utilizing gradient method. The smoothing process is performed in the input image *I*_*s*_ by applying Gaussian convolution filter. After that, gradient operation is applied to the image *I*_*G*_. The gradient of two variables *x* and *y* is defined as follows:

(1) $$\begin{array}{c} \nabla I_{G}\left(x,y\right)=\frac{\partial I_{G}}{\partial x}\overset{\wedge }{i}+\frac{\partial I_{G}}{\partial y}\overset{\wedge }{j}. \end{array}$$

Then, the binarization process is performed in the edge marked image *E*_*m*_ in [23]. The MRI brain image WM and GM tissues are segmented based on their intensity values [23]. The detailed process is given in [24]. Consider

(2) $$\begin{array}{c} I_{wg}=\left\{\begin{array}{cc} \text{WM}; & \text{if}\;I_{b_{i}}=1\\ \text{GM}; & \text{if}\;I_{b_{i}}=0. \end{array}\right. \end{array}$$

The results of WM and GM segmented images are denoted by *I*_*w*_, *I*_*g*_. Another one normal tissue CSF is segmented by the orthogonal polynomial transform (OPT). In orthogonal polynomial transformation, image *I*_*s*_ is computed using the following formula:

(3) $$\begin{array}{c} I_{cf}=\mathrm{Sin}{\left(\frac{{{I_{s}}_{\left(i\right)}}^{3}}{100}\right)}^{2}+\left(0.05*\text{rand}\left(\left|I_{s}\right|\right)\right). \end{array}$$

In (3), rand( ) represents a random value. After the polynomial transform, the corresponding CSF region is segmented in the resultant image *I*_*cf*_ [23].

#### 3.1.2. Abnormal Tissue Segmentation

Edema tissue is segmented from the abnormal image *I*_*s*_. Before the edema segmentation process, histogram equalization process is executed over the image *I*_*s*_. The quality of image *I*_*s*_ is enhanced by the histogram equalization and it is denoted by *I*_*s*_′. Then, the enhanced *I*_*s*_′ image is converted into indexed image by using multilevel thresholding function. Each pixel in the image is compared with these threshold values to select the pixels

(4) $$\begin{array}{c} H⟶t_{3},\;\;S⟶t_{4},\;\;V⟶t_{5}\\ X=\left\{\begin{array}{cc} p_{u}; & t_{4}\leq p_{u}\leq t_{3},t_{5}\\ 0; & \text{otherwise}. \end{array}\right. \end{array}$$

Subsequently, the distance is determined between the coordinates of the center pixels of the regions in *X*_*h*_^(*c*)^(*x*, *y*) and the tumor centroid coordinate value *t*(*x*, *y*)

(5) $$\begin{array}{c} O_{h}\left(x,y\right)=X_{h}^{\left(c\right)}\left(x,y\right)-t\left(x,y\right). \end{array}$$

The resultant *O*_*h*_(*x*, *y*) is then verified with threshold value *t*_6_ and edema region coordinate values are obtained

(6) $$\begin{array}{c} I_{e}=\left\{\begin{array}{cc} O_{h}\left(x,y\right)\geq t_{6} & \\ 0;\;\text{otherwise}. & \end{array}\right. \end{array}$$

Then, the morphological dilation and closing operations are performed in image *I*_*e*_.

The tumor tissue segmentation is performed in the abnormal brain MRI images. The main objective is to segment the tumor tissue in the abnormal image *I*_*s*_. Here, we utilize the region growing method (RGM) to segment the tumor tissue. Region growing method is a region based image segmentation method; it selects the initial seed points from the input image *I*_*s*_. The RGM observes the neighbor pixel values with the initial seed points, which checks whether the neighbor pixels are included in this region or not. The tumor segmentation result is represented as *I*_*t*_.

### 3.2. Feature Extraction

Training patterns are generated by extracting efficient features from the training MRI images. To accomplish the feature extraction in training and testing phase, the segmented images are utilized. The extracted features from the segmented images are discussed in Section 3.2.1.

#### 3.2.1. Feature Analysis

In this paper, we have utilized seven features. Among these seven features, two features are histogram based, two are statistical features, and the other three features are from wavelet. The individual performance of these features in the existing methods has both advantages and disadvantages. Thus, the combination of such features makes our proposed tissue segmentation method more efficient. All features are extracted from these nonzero blocks and computed the mean value for all feature values from each block

(7) $$\begin{array}{c} I_{i}^{c}=\overset{N}{\underset{k=1}{\sum }}I\left(b_{k}\right),\\ S_{i}^{c}=\overset{N}{\underset{k=1}{\sum }}S\left(b_{k}\right),\\ M_{i}^{c}=\overset{N}{\underset{k=1}{\sum }}M\left(b_{k}\right),\\ E_{i}^{c}=\overset{N}{\underset{k=1}{\sum }}E\left(b_{k}\right),\\ H_{i}^{c}=\overset{N}{\underset{k=1}{\sum }}H\left(b_{k}\right),\\ V_{i}^{c}=\overset{N}{\underset{k=1}{\sum }}V\left(b_{k}\right),\\ D_{i}^{c}=\overset{N}{\underset{k=1}{\sum }}D\left(b_{k}\right). \end{array}$$

The extracted features from the segmented images are represented as

(8) F=Iic,Sic,Mic,Eic,Hic,Vic,Dic,i=1,2,…I, c=1,2,…C,

where *i* is the number of segmented images and *c* is the segmented tissue classes. In (8), *I* denotes histogram intensity, *S* is the slope along the histogram, *M* is the mean value, *E* is the variance value, *H* denotes horizontal band, *V* denotes vertical band, and *D* is the diagonal band.

### 3.3. Heuristic Feature Selection by Improved Particle Swarm Optimization

Here, we utilize an improved PSO (IPSO) technique for improving the accuracy in classification process. The proposed IPSO technique considered both the bad and the good experience components during the new velocity computation. The bad experience component helps the particle to remember its previously visited worst position. To calculate the new velocity, the bad experience of the particle is also taken into consideration. On including the characteristics of *P* best and *P* worst in the velocity updating process along with the difference between the present best particle and current particle, respectively, the convergence towards the solution is found to be faster and an optimal solution is reached in comparison with conventional PSO approaches. Hence, our proposed IPSO selects the most optimal features from more number of features in the feature extraction process. More number of features in the training phase increase the time complexity and reduce the classification accuracy. To shun these drawbacks during the training process, we select the most optimal features from more number of available features. The selected optimal features from the ISPO technique are given to the FFBNN.

**Figure 1:**
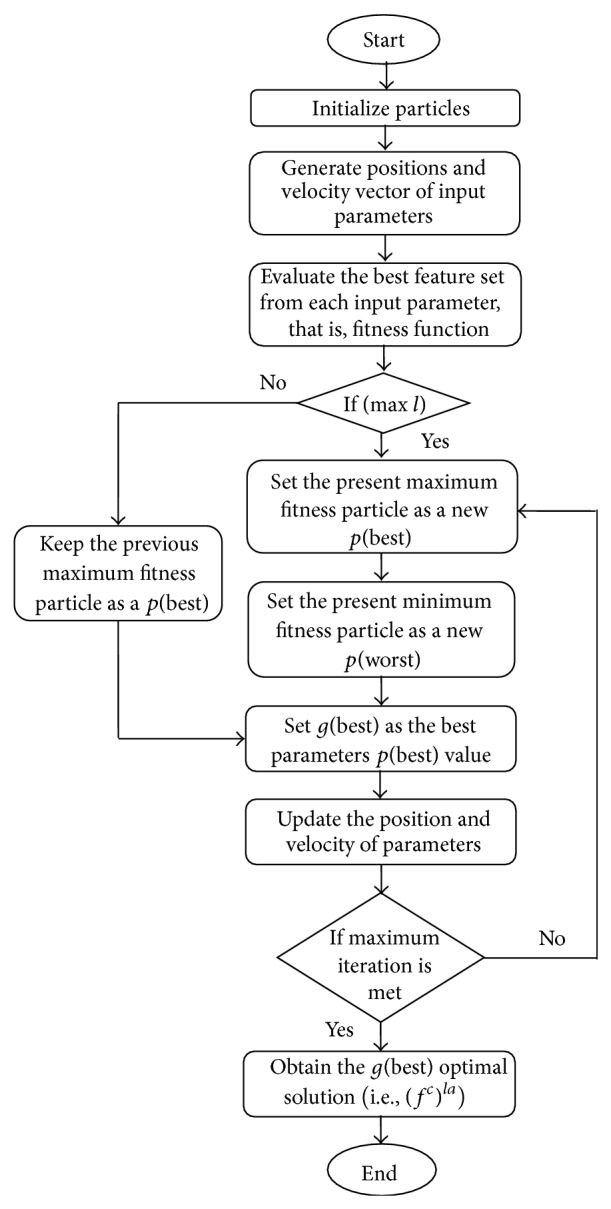
Flowchart of IPSO.

**Figure 2:**
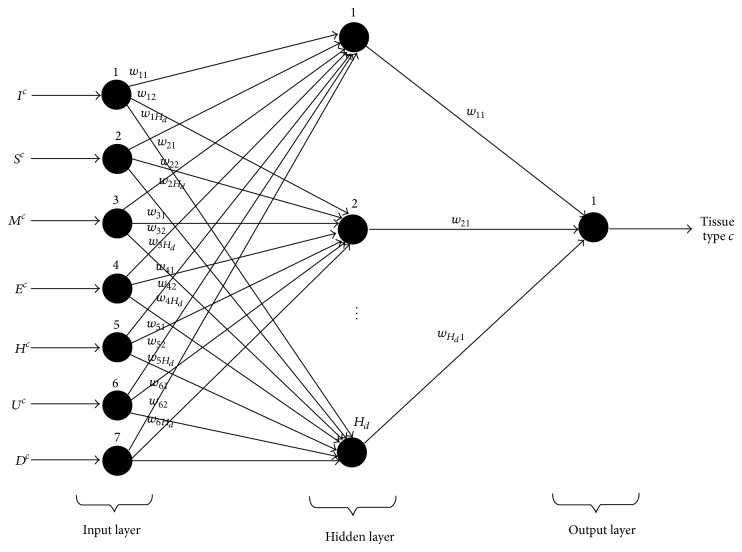
Proposed HGANN tissue classification *c*-FFBNN structure.

The process of optimal feature selection by IPSO technique is described as follows (Figure 1).

*(i) Initialization*. Initially the particles are generated for the class *c*. The defined particles are composed of the features, represented as *G* ^*l*^ = {*I* ^*c*^, *S* ^*c*^, *M* ^*c*^, *E* ^*c*^, *H* ^*c*^, *V* ^*c*^, *D* ^*c*^}, *c* = 1,2,…*C*.

*(ii) Parameters*. In IPSO, the particles position, velocity, learning parameters, inertia, weight, and maximum number of iterations are defined.

*(iii) Fitness*. Every particle's fitness value is calculated by using the formula which is given in (9). The particles that have the maximum fitness value are selected as the best particles

(9) fcla=Icl−Ica2+Scl−Sca2+Mcl−Mcanf−1  +Ecl−Eca+Hcl−Hca  Icl−Ica2+Vcl−Vca+Dcl−Dca·nf−11/2.

In (9),

  
*I* denotes histogram intensity;
  
*S* is the slope along the histogram;
  
*M* is the mean value;
  
*E* is the variance value;
  
*H* denotes the horizontal band;
  
*V* denotes the vertical band;
  
*D* is the diagonal band;
  
*l* is particle in the class *c*;
  
*a*′ is another particle in the class *c*;
  (*f* ^*c*^)^*la*^ is the best particles having the maximum fitness value.

The best particle denotes the maximum fitness value which means all features in the class *c* have high value. So, the tissue type is more accurately classified by selecting the maximum fitness value particles.

Select particles individual best value and particles global best value for each generation. In IPSO, we select the particles individual worst value, that is, the particle too away from the target.

*(iv) Velocity and Position*. Update particle individual best (*P* best), global best (*g* best), and particle worst (*P* worst) in the velocity formula which is given in the following equation and obtain the new velocity:

(10) Vi=w∗Vi+C1b∗r1∗Pbesti−Si∗Pbesti+C1w∗r2 ∗Si−Pworsti∗Pworsti+C2∗r3∗Gbesti−Si.

In (10),

  
*w* is the inertia weight;
  
*V*_*i*_ is the velocity of the particle;
  
*C*_1*b*_ is the acceleration coefficient in best position;
  
*C*_1*w*_ is acceleration coefficient in worst position;
  
*P*_best*i*_ is the best position of the particle *i*;
  
*S*_*i*_ is the current position of the particle;
  
*P*_worst*i*_ is the worst position of the particle *i*;
  
*r*_1_, *r*_2_, *r*_3_ are the uniformly distributed random numbers in the range [0 to 1].

Thus, the obtained new velocity value is updated in the original velocity formula given in (11). We obtain the position of the particle by the following:

(11) $$\begin{array}{c} V_{i}=w*V_{i}+C_{1}*r_{1}*\left(P_{\text{best}i}-S_{i}\right)+C_{2}*r_{2}*\left(G_{\text{best}i}-S_{i}\right), \end{array}$$

(12) $$\begin{array}{c} S_{i+1}=S_{i}+V_{i}. \end{array}$$

*(v) Stopping Criteria*. The process is repeated until the maximum number of iterations is reached. The final optimal feature set from IPSO technique is exploited in the tissues classification process.

### 3.4. Tissue Classification by FFBNN

**Figure 3:**
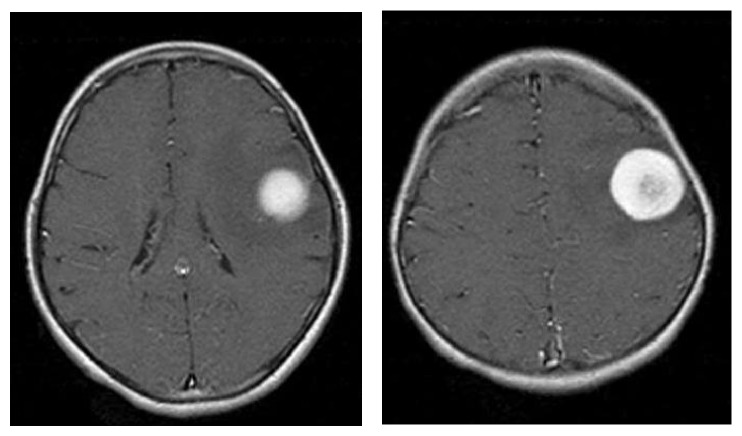
Sample input MRI brain images.

The feature set *F* ^*c*^ is given to the FFBNN classifier for training process and this classifier is represented as *c*-FFBNN. In the training phase, the selected heuristic features are given to the *c*-FFBNN network. The *c*-FFBNN network is well trained by these selected features. The network is created with seven input units from feature set *F* ^*c*^, that is, *F* ^*c*^ = {*I* ^*c*^, *S* ^*c*^, *M* ^*c*^, *E* ^*c*^, *H* ^*c*^, *V* ^*c*^, *D* ^*c*^}, *H*_*d*_ hidden units, and one output unit, that is, *c*. The basic structure of FFBNN network is shown in Figure 3. This FFBNN is used for classifying the input feature set that belongs to which tissue class type.

The following steps portray the function of the neural network.

**Step 1 .**

Assign input weights to all neurons except the neurons in the input layer. The planned bias function and activation function for the neural network are described as follows:

(13) Zc=β+∑n′=0Hd−1wcn′In′c+wcn′Sn′c+wcn′Mn′c+wcn′En′c     +wcn′Hn′c+wcn′Vn′c+wcn′Dn′c,δXc=11+e−Zc.

Equation (10) represents the input layer bias function. Here, (*I*_*n*′_^*c*^), (*S*_*n*′_^*c*^), (*M*_*n*′_^*c*^), (*E*_*n*′_^*c*^), (*H*_*n*′_^*c*^), (*V*_*n*′_^*c*^), and (*D*_*n*′_^*c*^) are the features of the class *c*. Equation (11) represents the activation function (*δ*) for the output layer.

**Step 2 .**

Calculate the learning error for the neural network

(14) $$\begin{array}{c} L^{\left(e\right)}=\frac{1}{H_{d}}\overset{H_{d}-1}{\underset{n^{\prime }=0}{\sum }}D_{n^{\prime }}-Z_{n^{\prime }}. \end{array}$$

In (14), *L* ^(*e*)^ is the learning error rate and *D*_*n*′_ and *Z*_*n*′_ are the desired and actual outputs, respectively. The error that occurred during the FFBNN training process is minimized by the back propagation algorithm. The error minimization process by back propagation algorithm is briefly explained in [24]. The *c*-FFBNN network is well trained by these selected features and this network classifies the particular feature values based on the tissue types it belongs or not. Similar FFBNN training process is carried out for the all feature set training.

In testing phase, a new set of testing images are taken for the classification. The testing images normal and abnormal tissues are segmented by the method, which is described in Section 3.1. From the segmented images, the features M, V, VB, DB, HB, Hist-Inten, and Hist-Slope values are calculated and these extracted feature values are given as input to the well-trained *c*-FFBNN classifiers. Based on the input features, the specific tissue type *c*-FFBNN provides any one of the tissue types such as WM, GM, CSF, tumor, or edema as output. In this manner, the MRI brain image tissues are classified.

## 4. Experimental Results and Discussion

The proposed MRI brain tissues classification technique is implemented in the working platform of MATLAB (version 7.12) with machine configuration as follows:

 processor: Intel core i5;
 OS: Windows 7;
 CPU speed: 3.20 GHz;
 RAM: 4 GB.

**Figure 4:**
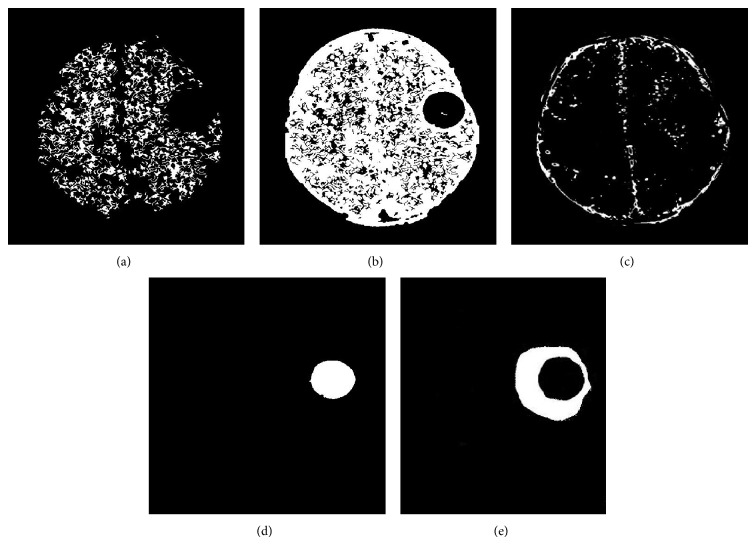
Manually segmented MRI brain tissues: (a) WM, (b) GM, (c) CSF, (d) tumor, and (e) edema.

The brain MRI images are collected from various medical diagnosis centers; thus, the collected MRI sample images and segmented results are shown in Figures 3 and 4.

From these manually segmented images, seven features such as histogram intensity, slope of histogram, mean, variance, horizontal, vertical, and diagonal functions of 2D wavelet decomposition are extracted, and, using these extracted features, the heuristic features are generated for sample 5 images by IPSO given in Table 1.

**Figure 5:**
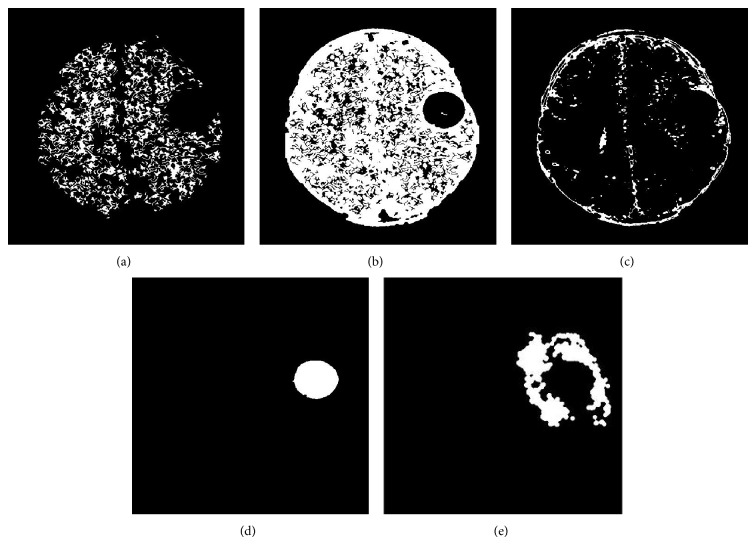
Segmented MRI brain tissues: (a) WM, (b) GM, (c) CSF, (d) tumor, and (e) edema.

Utilizing these heuristic features, the FFBNN are well trained. In testing process, the testing image's normal and abnormal tissues are segmented by the methods in [23]. The segmented image results are shown in Figure 5.

*Performance Analysis*. The performance of proposed tissue classification method is analyzed by the statistical measures. The normal and abnormal tissues classification accuracy is calculated by these statistical measures, which are shown in Table 2.

As can be seen from Table 2, there are three images utilized in the performance analysis. The classification of normal WM, GM, and CSF tissues has given 93%, 87%, and 93% mean accuracy results, respectively. Thus, this higher accuracy has offered more precise classification in the normal images. Furthermore, the pathological tissues such as edema and tumor have also given 87% and 93% mean accuracy results, respectively. Hence, our proposed tissue classification method has offered more efficient and effective results in both normal and pathological tissues classification processes.

*Comparative Analysis*. The classification performance of proposed IPSONN is analyzed with the existing tissue classification methods [22, 24]. This existing tissue classification method has utilized FRBS and hybrid genetic approach. The existing classification methods results are shown in Tables 3 and 4.

**Figure 6:**
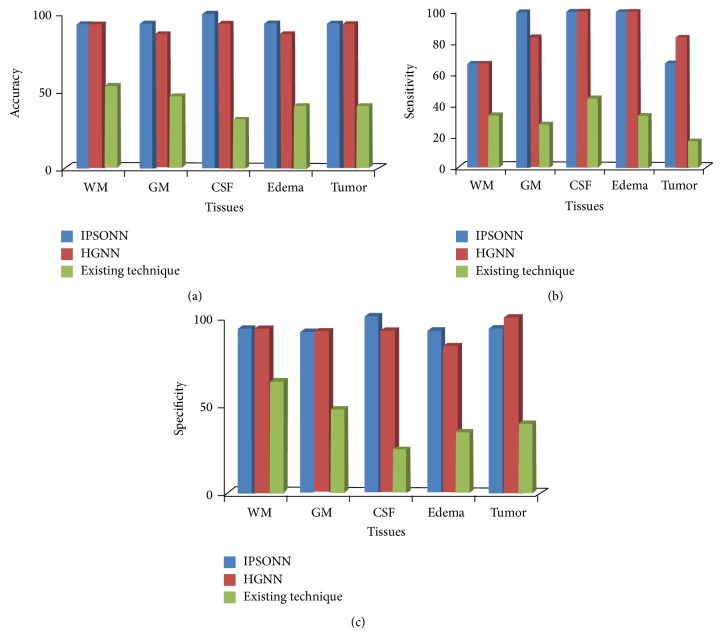
Tissues classification result: (a) accuracy, (b) sensitivity, and (c) specificity.

The graphical representation of the proposed and existing techniques average performance in tissue classification process is shown in Figure 6.

Figure 6 shows the graphical representation of WM, GM, CSF, tumor, and edema tissue classification performance compared to the existing HGNN and hybrid genetic method. As can be seen from Figure 6(a), the existing hybrid approach has given lower accuracy than the methods HGNN and IPSONN. When compared to HGNN and IPSONN, our proposed IPSONN has given higher accuracy in GM, CSF, and edema than HGNN, but the other two tissues accuracy results are the same for both methods. The tissues classification methods IPSONN, HGNN, and the existing hybrid genetic approach attain an overall mean accuracy of 95%, 91%, and 42%, respectively. Our proposed IPSONN tissues classification method has given higher accuracy result than the existing methods. In Figures 6(b) and 6(c), HGNN attains high sensitivity and specificity in tumor tissues classification, but the other tissues classification our proposed method maintains high and same sensitivity and specificity level than the HGNN and Existing hybrid approach. In sensitivity performance review, our proposed IPSONN and HGNN attain 87% and the existing hybrid genetic approach conquers only 31% sensitivity. In specificity measure, the tissues classification methods like IPSONN, HGNN, and hybrid genetic approach achieve 94%, 92%, and 42% specificity, respectively. Hence, our proposed IPSONN has given high performance in tissues classification than the HGNN and the existing hybrid approach.

## 5. Conclusion

In this paper, we proposed a new tissue classification method called IPSONN to classify the normal and abnormal tissues from the MRI images. The method was implemented and a smaller set of MRI brain images were utilized to analyze the results of the IPSONN classification method. In future works, the results presented in this paper should be extended and verified using larger and diverse image datasets. The performance analysis proved that the IPSONN method offers almost 95% accuracy and 87% and 94% (in average) sensitivity and specificity measures, respectively. The IPSONN method was compared against the existing tissues classification methods to prove the performance. Thus, the results show that the IPSONN achieved more classification performance than the existing hybrid genetic and HGNN methods.

## Figures and Tables

**Table 1:** Heuristic features from IPSO.

Heuristic features from IPSO							
Images	Histogram intensity	Slope of histogram	Mean	Variance	Horizontal value of 2D wavelet decomposition	Vertical value of 2D wavelet decomposition	Diagonal value of 2D wavelet decomposition
1	2	255	1.049225	28.47425	22.17121	8.785642	11.08208
2	2	255	2.263733	66.02611	31.9345	13.95081	6.356591
3	2	255	1.886505	48.81538	23.90411	21.98224	20.34238
4	2	255	1.629013	38.28219	12.74081	18.08271	18.13534
5	2	255	2.083221	63.93179	27.44711	26.96236	19.40522

**Table:** (a)

Images	TP	FP	TN	FN	Sensitivity	FPR	ACC	Specificity	PPV	NPV	FDR	MCC
1	1	0	4	0	100	0.0	100	100	100	100	0	44.7
2	0	1	4	0	0	20.0	80	80	0	100	100	0
3	1	0	4	0	100	0.0	100	100	100	100	0	44.7

**Table:** (b)

Images	TP	FP	TN	FN	Sensitivity	FPR	ACC	Specificity	PPV	NPV	FDR	MCC
1	1	1	3	0	100	25.0	80	75	50	100	50	43.3
2	1	0	4	0	100	0.0	100	100	100	100	0	44.7
3	1	0	4	0	100	0.0	100	100	100	100	0	44.7

**Table:** (c)

Images	TP	FP	TN	FN	Sensitivity	FPR	ACC	Specificity	PPV	NPV	FDR	MCC
1	1	0	4	0	100	0.0	100	100	100	100	0	44.7
2	1	0	4	0	100	0.0	100	100	100	100	0	44.7
3	0	1	4	0	100	0.0	100	100	100	100	0	44.7

**Table:** (d)

Images	TP	FP	TN	FN	Sensitivity	FPR	ACC	Specificity	PPV	NPV	FDR	MCC
1	1	0	4	0	100	0.0	100	100	100	100	0	44.7
2	1	1	3	0	100	25.0	80	75	50	100	50	43.3
3	1	0	4	0	100	0.0	100	100	100	100	0	44.7

**Table:** (e)

Images	TP	FP	TN	FN	Sensitivity	FPR	ACC	Specificity	PPV	NPV	FDR	MCC
1	0	1	4	0	0	20.0	80	80	0	100	100	0
2	1	0	4	0	100	0.0	100	100	100	100	0	44.7
3	1	0	4	0	100	0.0	100	100	100	100	0	44.7

**Table:** (a)

Images	TP	FP	TN	FN	Sensitivity	FPR	ACC	Specificity	PPV	NPV	FDR	MCC
1	0	1	3	1	0	25.0	60	75	0	75	100	−14.4
2	1	2	2	0	100	50.0	60	50	33	100	67	40.8
3	0	1	2	2	0	33.3	40	67	0	50	100	−28.9

**Table:** (b)

Images	TP	FP	TN	FN	Sensitivity	FPR	ACC	Specificity	PPV	NPV	FDR	MCC
1	1	1	1	2	33	50.0	40	50	50	33	50	−16.7
2	0	2	3	0	0	40.0	60	60	0	100	100	0
3	1	2	1	1	50	66.7	40	33	33	50	67	−20.4

**Table:** (c)

Images	TP	FP	TN	FN	Sensitivity	FPR	ACC	Specificity	PPV	NPV	FDR	MCC
1	0	3	2	0	0	60.0	40	40	0	100	100	0
2	1	2	1	2	33	66.7	33	33	33	33	67	−40.8
3	1	4	0	0	100	100.0	20	0	20	0	80	0

**Table:** (d)

Images	TP	FP	TN	FN	Sensitivity	FPR	ACC	Specificity	PPV	NPV	FDR	MCC
1	0	3	2	0	0	60.0	40	40	0	100	100	0
2	0	3	2	0	0	60.0	40	40	0	100	100	0
3	1	3	1	0	100	75.0	40	25	25	100	75	35.4

**Table:** (e)

Images	TP	FP	TN	FN	Sensitivity	FPR	ACC	Specificity	PPV	NPV	FDR	MCC
1	1	2	1	1	50	66.7	40	33	33	50	67	−20.4
2	0	3	1	1	0	75.0	20	25	0	50	100	−106.1
3	0	2	3	0	0	40.0	60	60	0	100	100	0

**Table:** (a)

Images	TP	FP	TN	FN	Sensitivity	FPR	ACC	Specificity	PPV	NPV	FDR	MCC
1	1	0	4	0	100	0.0	100	100	100	100	0	44.7
2	1	0	4	0	100	0.0	100	100	100	100	0	44.7
3	0	1	4	0	0	20.0	80	80	0	100	100	0

**Table:** (b)

Images	TP	FP	TN	FN	Sensitivity	FPR	ACC	Specificity	PPV	NPV	FDR	MCC
1	1	1	3	0	100	25.0	80	75	50	100	50	43.3
2	1	0	3	1	50	0.0	80	100	100	75	0	30.6
3	1	0	4	0	100	0.0	100	100	100	100	0	44.7

**Table:** (c)

Images	TP	FP	TN	FN	Sensitivity	FPR	ACC	Specificity	PPV	NPV	FDR	MCC
1	1	0	4	0	100	0.0	100	100	100	100	0	44.7
2	1	1	3	0	100	25.0	80	75	50	100	50	43.3
3	1	0	4	0	100	0.0	100	100	100	100	0	44.7

**Table:** (d)

Images	TP	FP	TN	FN	Sensitivity	FPR	ACC	Specificity	PPV	NPV	FDR	MCC
1	1	1	3	0	100	25.0	80	75	50	100	50	43.3
2	1	0	4	0	100	0.0	100	100	100	100	0	44.7
3	1	1	3	0	100	25.0	80	75	50	100	50	43.3

**Table:** (e)

Images	TP	FP	TN	FN	Sensitivity	FPR	ACC	Specificity	PPV	NPV	FDR	MCC
1	1	0	3	1	50	0.0	80	100	100	75	0	30.6
2	1	0	4	0	100	0.0	100	100	100	100	0	44.7
3	1	0	4	0	100	0.0	100	100	100	100	0	44.7

## References

[1] Zhu C., Jiang T. (2003). Multicontext fuzzy clustering for separation of brain tissues in magnetic resonance images. *NeuroImage*.

[2] Shen S., Sandham W., Granat M., Sterr A. (2005). MRI fuzzy segmentation of brain tissue using neighborhood attraction with neural-network optimization. *IEEE Transactions on Information Technology in Biomedicine*.

[3] Sutar M., Janwe N. J. (2011). A swarm-based approach to medical image analysis. *Global Journal of Computer Science and Technology*.

[4] Maji P., Kundu M. K., Chanda B. Segmentation of brain MR images using fuzzy sets and modified co-occurrence matrix.

[5] Senthilkumaran N., Rajesh R. (2009). Brain image segmentation using granular rough sets. *International Journal of Arts & Sciences*.

[6] Forghani N., Forouzanfar M., Forouzanfar E. MRI fuzzy segmentation of brain tissue using IFCM algorithm with particle swarm optimization.

[7] Revett K., Khan A. An on-line (real-time) automated MRI based pathology detection system using selforganised maps.

[8] Maji P., Kundu M. K., Chanda B. (2008). Second order fuzzy measure and weighted co-occurrence matrix for segmentation of brain MR images. *Fundamenta Informaticae*.

[9] Mostafa M. G., Tolba M. F., Gharib T. F., A-Megeed M. A Gaussian multiresolution algorithm for medical image segmentation.

[10] Lin J. S., Cheng K. S., Mao C. W. (1996). Segmentation of multispectral magnetic resonance image using penalized fuzzy competitive learning network. *Computers and Biomedical Research*.

[11] Rajapakse J. C., Giedd J. N., Rapoport J. L. (1997). Statistical approach to segmentation of single-channel cerebral MR images. *IEEE Transactions on Medical Imaging*.

[12] Wells W. M., Crimson W. E. L., Kikinis R., Jolesz F. A. (1996). Adaptive segmentation of MRI data. *IEEE Transactions on Medical Imaging*.

[13] Ibraheem Jabbar N., Mehrotra M. (2008). Application of fuzzy neural network for image tumor description. *World Academy of Science, Engineering and Technology*.

[14] Karayiannis N. B. (1997). A methodology for constructing fuzzy algorithms for learning vector quantization. *IEEE Transactions on Neural Networks*.

[15] Karayiannis N. B., Pai P.-I. (1999). Segmentation of magnetic resonance images using fuzzy algorithms for learning vector quantization. *IEEE Transactions on Medical Imaging*.

[16] Ishibuchi H., Nakashima T., Murata T. (2001). Three-objective genetics-based machine learning for linguistic rule extraction. *Information Sciences*.

[17] Admasu F., Al-Zubi S., Toennies K., Bodammer N., Hinrichs H. Segmentation of multiple sclerosis lesions from MR brain images using the principles of fuzzy-connectedness and artificial neuron networks.

[18] Subbanna N. K., Shah M., Francis S. J. MS lesion segmentation using Markov random fields.

[19] Wang C.-M., Chen R.-M. (2011). Vector seeded region growing for parenchyma classification in brain MRI. *International Journal of Advancements in Computing Technology*.

[20] Cherradi B., Bouattane O., Youssfi M., Raihani A. (2011). Fully automatic method for 3D T1-weighted brain magnetic resonance images segmentation. *International Journal of Image Processing*.

[21] Rajendran A., Dhanasekaran R. (2011). MRI brain image tissue segmentation analysis using possibilistic fuzzy C-means method. *International Journal on Computer Science and Engineering*.

[22] Mehta S. B., Chaudhury S., Bhattacharyya A., Jena A. (2011). Tissue classification in magnetic resonance images through the hybrid approach of Michigan and Pittsburg genetic algorithm. *Applied Soft Computing Journal*.

[23] Hussain J., Savithri S., Devi S. (2012). Segmentation of tissues in brain MRI images using dynamic neuro-fuzzy technique. *International Journal of Soft Computing and Engineering*.

[24] Sheejakumari V., Gomathi A. (2012). Healthy and pathological tissues classification in MRI brain images using hybrid genetic algorithm-neural network (HGANN) approach. *European Journal of Scientific Research*.

